# Meta-Analysis of the Effect of Yoga Practice on Physical Fitness in the Elderly

**DOI:** 10.3390/ijerph182111663

**Published:** 2021-11-06

**Authors:** Sohee Shin

**Affiliations:** School of Sport and Exercise Science, University of Ulsan, 93 Daehak-ro, Nam-gu, Ulsan 44610, Korea; soheeshin@ulsan.ac.kr

**Keywords:** yoga intervention, the elderly, physical fitness

## Abstract

The purpose of this study was to meta-analyze the effects of yoga intervention on physical fitness in the elderly. The following databases were systematically searched in 25 March 2021: Cochrane, PubMed and Embase. A total of 656 papers was identified through key word combinations, finally, 12 studies were included in the meta-analysis. The main conclusions are as follows. First, yoga practice showed moderately positive effects on muscle strength, balance, mobility, and lower body flexibility, but had no significant effect on cardiorespiratory endurance and upper body flexibility. Second, sub-group analysis showed that subjects in their 60s and 70s and yoga practice for 9–12 weeks had a large positive effect on physical fitness. Yoga is a multimodal activity that improves muscle strength, balance, and flexibility in the elderly, and physical activity policies should continue to promote yoga as an activity that enhances physical and mental wellbeing in this population.

## 1. Introduction

An important goal for most older people is to lead an independent life. Yoga is known to have various health benefits including: (1) lowered blood glucose for people with type 2 diabetes; (2) improved symptoms of depression and anxiety; (3) decreased pain; (4) improved sleep disturbance; and (5) improved quality of life. Yoga integrates physical, mental, and emotional dimensions to promote health. Many people prefer adopting yoga practice because it is easy to learn, can be performed effectively in a narrow space without special equipment, and can be practiced by various age groups to manage health [1]. On the other hand, some studies have reported that the amount of physical activity in yoga practice does not meet the recommendations for the level of physical activity to promote cardiovascular health, and that it does not affect body composition [1]. Although various research results related to the effects of yoga practice are accumulating, it is difficult to derive comprehensive yoga usefulness because of the variability in training content of these studies and the effects on physical health-related variables. Accordingly, there is a need to synthesize the effects of yoga practice on physical health through meta-analysis [1].

As data from various studies have accumulated, meta-analysis studies on the effects of yoga practice have been steadily progressing. Various yoga studies focusing on the exercise effect of yoga, pain relief for rheumatoid osteoporosis [2,3], cardiovascular function improvement [4,5], positive effect on cognitive function and mental health [6,7,8,9], diabetes improvement [10,11], blood pressure lowering [12,13], side effect improvement of breast cancer [14], and the effect of Thai yoga and laughter yoga on physical fitness [15,16] have been reported.

While previous meta-analyses tried various approaches, meta-analysis on the effects of various types of yoga training on physical fitness of the elderly has not been reported. Physical fitness of the elderly is related closely to physical health, risk of falls, and ability to perform independent daily activities. Therefore, a comprehensive review of the effects of yoga training on physical fitness can provide a basis for the composition and effective operation of yoga training programs for the elderly.

## 2. Materials and Methods

### 2.1. Search and Selection Criteria

**Figure 1:**
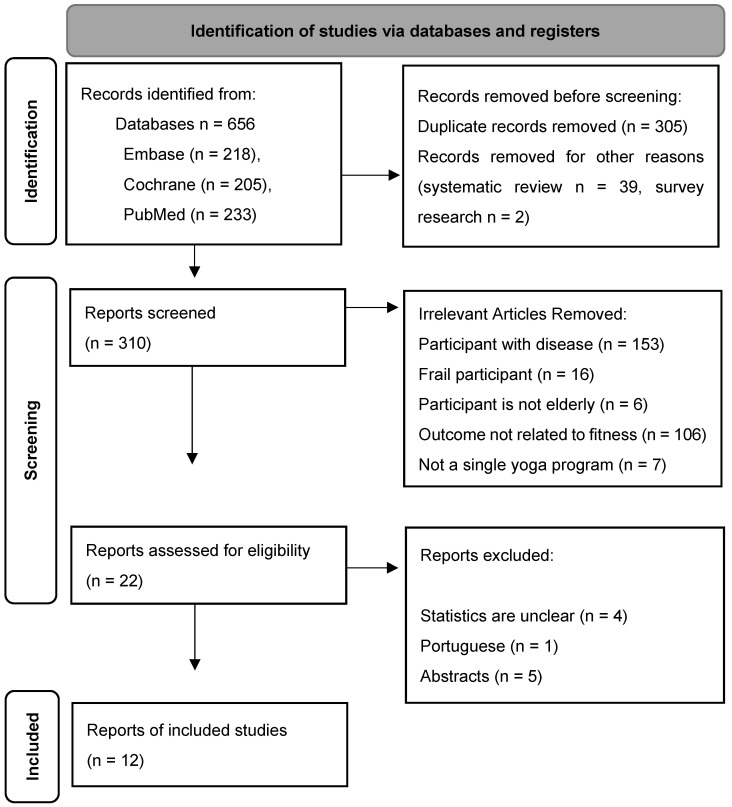
Elimination process of the articles selected for the review (PRISMA 2020 flow diagram [19]).

This review was conducted in accordance with Preferred Reporting Items for Systematic Reviews and Meta-Analyses (PRISMA) guidelines [17] and recommendations of the Cochrane collaboration [18]. The protocol was developed in advance of the study and registered on PROSPERO (Registration number: CRD42021247535). Search criteria were established to select systematically research papers. First, study subjects were selected as elderly people over 60 years of age. Second, the intervention method was limited to a single yoga training program. Third, as a result of the intervention, the physical fitness variable was selected as the dependent variable. Fourth, the research design was limited to an experimental design in the form of results comparison through pre–post tests. Fifth, studies were selected in which specific statistical values for calculation of effect size were presented. The main search terms used in this study included yoga, the elderly, and RCT research. The Cochrane, Embase, and PubMed databases were used for literature searches. The paper search was conducted on 25 March 2021. A total of 656 papers was identified through key word combinations. Finally, 12 studies were included in the meta-analysis. The elimination process of the articles selected for the review is shown in the PRISMA flow chart in Figure 1, and the characteristics of the finally selected study to be analyzed are shown in Table 1. Two investigators independently selected studies for inclusion; disagreements were resolved by discussion.

### 2.2. Coding

Coding was carried out after writing a coding book for the characteristics of the literature to be analyzed. Coding items were entered by author (year of publication), participants, number of cases in the group, age, intervention period, and outcome variable (statistical value for each physical fitness variable). The classification of the result parameters was systematized through two steps. In step 1, the parameters were divided by physical fitness factor. Physical fitness was categorized as upper limb strength, lower limb strength, upper body flexibility, lower body flexibility, mobility, cardio-respiratory fitness, and balance. In step 2, similar result parameters were categorized as the same factors to minimize the amount of information that was lost when analyzing each parameter.

### 2.3. Analysis Methods

The coded data were analyzed using the CMA (Comprehensive Meta-Analysis) 3.0 program. First, the effect of each result parameter was calculated as the Cohen’s d. ES < 0.40 was a small effect size, ES = 0.40–0.70 means a moderate effect size, and ES > 0.70 means a large effect size (Cohen, 1988). Second, Q and I^2^ were calculated to verify heterogeneity of the effect size. If the Q value is less than 0.1, there is possible heterogeneity of effect size. The heterogeneity of I^2^ can be interpreted as follows; 0–25% is low, 25–49% is medium, 50–74% is high, and 75–100% is quite high (Higgins, 2008). If homogeneity was determined, the fixed-effect model was used; if heterogeneity was determined, random-effect model analysis was used. Third, to verify publication bias in this study, the symmetry of data was reviewed through a funnel plot.

## 3. Results

### 3.1. Homogeneity Test and Total Effect Size

Results of the homogeneity test related to the effect of yoga intervention on physical fitness are shown in Table 2. Statistical significance of the Q value was smaller than 0.000, and I2 was 67.1; therefore, the heterogeneity was greater than moderate, and it was assumed that the articles targeted in this study were not homogeneous as analyzed using the Random-Effect Model. As a result, the effect size was moderate at 0.518 by the Cohen [32].

### 3.2. Effect Size for Each Physical Fitness Factor

The average effect size of the physical fitness factor was significant (Table 3). Yoga intervention showed the largest effect on upper limb strength (ES = 0.65). The effects of yoga intervention on balance (ES = 0.64), mobility (ES = 0.59), lower limb strength (ES = 0.55), and lower body flexibility (ES = 0.49) were moderate. However, the effects of yoga intervention on cardiopulmonary endurance and upper body flexibility were low.

### 3.3. Effect Size for Each Age Factor

The average effect size of the age factor was significant (Table 3). The effect size of yoga intervention for subjects in their 60s (ES = 0.58) and 70s (ES = 0.56) was moderate. However, the effect of yoga intervention for subjects in their 80s (ES = 0.17) was low.

### 3.4. Effect Size for Each Period Factor

The average effect size of the period factor was significant (Table 3). The effect size of yoga intervention for the 9–12 week period (ES = 0.70) was high. The period shorter than 8 weeks had moderate ES (0.54). However, the effects of yoga intervention on the period over 13 weeks was low (ES = 0.23).

### 3.5. Publication Bias

**Figure 2:**
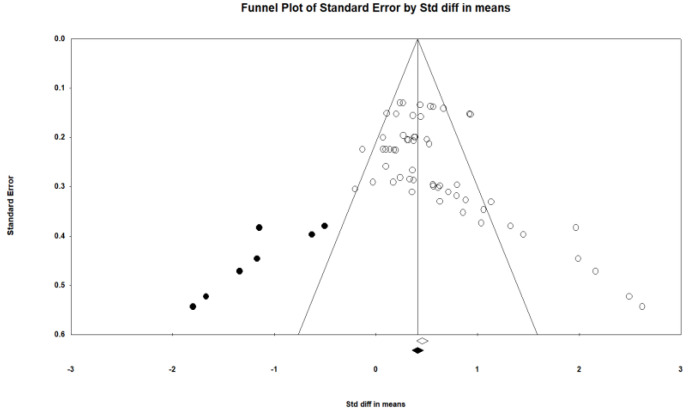
Funnel plot.

For all physical fitness parameters, asymmetry was found in the funnel plot (Figure 2), indicating that the larger the standard error (SE), the larger the ES. Egger’s regression intercept was performed to statistically examine asymmetry between SE and ES. As a result, the value for the fragment was significant, indicating a publication bias (T = 3.50, *p* < 0.05). Using Trim and Fill, seven studies assumed to be missing were used to correct asymmetry, and the effect size of 0.45 (95% CI: 0.40–0.51) before calibration decreased to 0.41 (95% CI: 0.35–0.47) after correction but was still significant.

## 4. Discussion

The study conducted was a meta-analysis on the effect of yoga intervention on physical fitness of the elderly. The implications of the main results are discussed as follows.

First, the overall effect size of yoga intervention on physical fitness was ES = 0.518, suggesting that yoga practice can have a positive effect on physical fitness in the elderly. Many studies for young people and adults reported that yoga increases muscle strength, power, endurance, flexibility, and balance and coordination in young people [25]. The elderly participants did not show much improvement in physical fitness through exercise compared to young people, and the aspect of maintaining physical fitness was emphasized rather than improving physical fitness. Additionally, considering that yoga is a low-to-moderate intensity exercise compared to other exercises, the result that yoga had a positive effect on improving physical fitness of the elderly is meaningful. Tew, Howsam [29] also reported that the proposed benefits of regular yoga practice for the elderly are numerous and varied, including increases in muscular strength, flexibility and balance, reduced stress, anxiety and depression, and an enhancement of overall well-being and quality of life.

Among the physical fitness factors, upper limb strength and balance showed a high effect size (0.6 or more), and mobility, lower limb strength, and lower body flexibility showed a moderate effect size (0.5). People who have poor grip strength, which is used as an indicator of whole-body strength, or the ability to get up from a chair, have a high risk of death [33]. In particular, decreased lower limb strength impairs motor skills such as walking, climbing stairs, getting up from a chair, and balancing [34], and increases the risk of falls and hip fractures [35,36]. In the West, the word ‘yoga’ is the general term used for the practice of ‘Hatha Yoga’. Hatha Yoga is a centuries-old health and well-being system from India that involves a combination of physical postures or poses (asana), breathing exercises (pranayama), integrated breath-movement sequences, relaxation, and concentration/meditation [29]. Improvements in physical measures directly related to yoga intervention are not surprising. Yoga practice involves training on poses very similar to these outcome measures [28].

There are various types of yoga asana, and the elderly can use support tools such as straps, blocks, bolsters, pillows, and chairs; but basically, participants do not use special equipment and use their own body weight to pose on a mat. Yoga exercise is practiced through muscle relaxation, and it improves flexibility through repetition of tension and relaxation [37]. In addition, since most postures require balance and muscle strength, such as standing balance posture, table posture, and posture supporting weight with both arms and two feet while raising the hips [37], it is judged to be helpful in improving muscle strength and balance. Mobility is the ability that the elderly need for independent daily life. It requires not only strength, balance, and flexibility, but also coordination, and is used widely as a tool for screening fall risk in the elderly [38,39]. As a result of the meta-analysis, improved mobility would be closely related to improvement of muscle strength, balance, and flexibility. 

As a result of sub-group analysis of ‘time period’ as a moderating variable, yoga for 9–12 weeks was the most effective for improving physical fitness. Exercise is performed by appropriately prescribing form, intensity, and time frequency according to purpose, but the effect can be obtained only when it is performed regularly and over a certain period of time. Temporary or short-term exercise effects can return to the original state in a short period of time, and if a similar exercise type program is implemented for too long, exercise program participation rate and interest in exercise in the participants can decrease. Tiedemann, O’Rourke [30] demonstrates that the yoga program needs to be appropriate to the abilities of the older participants and also enjoyable, with participants reporting perceived benefits as a result of their attendance, and adherence was promoted by cohesive group dynamics, gradual increases in program intensity, and strong instructor leadership.

Summarizing the above, it would be effective to practice yoga for a period of 9–12 weeks based on the exercise type, exercise intensity, and instructor leadership suitable for participant physical condition in order to improve physical fitness of the elderly. This is because the frequency of group yoga practice was 1–3 times a week, but most studies recommended that participants do the exercise at home as well, although the exact number was not counted. However, it was judged that the exercise performed at home did not have a significant effect on the exercise result because a few simple movements were performed differently from the group exercise.

As a result of examining the effect of yoga training on physical fitness with ‘age’ as a moderating variable, the lower the age, the higher the effectivity (60s > 70s > 80s). The ES was 0.58 for subjects in their 60s and 0.56 for those in their 70s, and although the 60s were slightly higher than the 70s, they were effective at a similar level, but there was no significant effect on improving physical fitness for subjects in their 80s. Even the older elderly in their 80s with a low physical fitness level can practice yoga using tools such as chairs, belts, and bands to suit his or her physical condition. However, the elderly in their 80s are focused on maintaining physical fitness rather than improving physical fitness, and the yoga movements they can perform are limited and low in intensity, so it would not have been possible to improve their physical fitness. However, senior yoga is known to have the effect of improving symptoms of depression and anxiety, decreasing pain, and improving sleep disturbance through regulation of the breath (pranayama) and relaxation (meditation) as well as maintaining physical fitness [40]. Additionally, yoga programs are safe for the elderly and have few side effects, so it is highly recommended for the elderly. Yoga is a multimodal activity that improves muscle strength, balance, and flexibility in the elderly [40], and physical activity policies should continue to promote yoga as an activity that enhances physical and mental wellbeing in this population.

## 5. Limitations in Research

The results of this study are meaningful in that they analyzed the effects of yoga training, which has recently been attracting attention, on physical fitness using factors such as physical fitness, age, and period as moderating variables. Nevertheless, this study has the following limitations that indicate suggestions for further research. First, the frequency according to period was not included as a moderating variable in this study, and the moderating variable was set as a categorical variable, which is an arbitrary variable. As previous studies referenced for category classification are limited, different results can be observed depending on the category classification method. In follow-up studies, it is necessary to try various analyses by referring to more diverse prior data. Second, in the papers analyzed in this study, information on the type of yoga practice was not presented clearly in many cases, so the effect analysis according to yoga type was excluded. In the future, effect analysis should be conducted focusing on studies that include information on yoga type. Third, because publication bias appeared in this study, it is necessary to include not only RCT studies but also various publication types of papers in the analysis. Fourth, there was no prospective study on changes in physical fitness after yoga practice in this study, it would be interesting how the physical fitness changes some time after completing 9–12 weeks of yoga exercise.

## 6. Conclusions

This study comprehensively analyzed the effects of yoga practice on physical fitness by targeting related studies through meta-analysis. The main conclusions drawn from the study results are as follows. First, yoga practice showed moderately positive effects on muscle strength, balance, mobility, and lower body flexibility, but had no significant effect on cardiorespiratory endurance and upper body flexibility. Second, sub-group analysis showed that subjects in their 60s and 70s and yoga practice for 9–12 weeks had a large positive effect on physical fitness.

## Figures and Tables

**Table 1:** Characteristics of research papers.

No.	Study	Region	Design	Participants	Intervention Methods	Main Outcome Measures
**1**	Bucht et al., 2019 [20]	Germany	RCT	Healty and active older adults, n: men 1 and women 10, age: 68.7 yrs	Sauna yoga, 30 min, 1/week × 8week	Chair sit and reach, Back scratch, Lateral flexion, 5 times STS, Sharpened Romberg eyes open/eyes closed
**2**	Furtado et al., 2016 [21]	Portugal	RCT	Older women living in social and health care support centres, n: women 20, age: 83.81 yrs	Chair yoga, 2–3/week × 14 week	Chair sit and reach, 30 s arm curl test, 30 s chair and stand test, 8 foot up and go test
**3**	Gothe et al., 2016 [22]	USA	RCT	Sedentary healthy adults n: men 12 and women 49, age: 62.1 yrs	Hatha yoga(use a belt), 60 min, 3/week × 8 week	Balance, Four square step, 30 s Arm curls, 30 s chair and stand test, 4m gait speed, 8 feet up and go test, Back scratch, Sit and reach
**4**	Marques et al., 2017 [23]	Portugal	RCT	Older women living in social caregiver center, n: women 15, age: 83.7 yrs	Chair yoga, 55 min, 2–3/week × 28 week	2 min step test, 8 feet up and go test, Chair sit and reach
**5**	Ni et al., 2014 [24]	USA	RCT	60 years of age or older, living indpendently, n: men 3 and women 10, age: 73.2 yrs	Vinyasa yoga(focus on balance), 60 min, 2/week × 12 week	8 feet up and go test, One Leg Stand, Functional Reach, usual walking, max walking
**6**	Nick et al., 2016 [25]	Iran	RCT	Older adults, n: men 9 and women 11, age: 68 yrs	Hatha yoga (Focus on pavanamuktasana and balance), 60 min, 2/week × 8 week	Berg balance scale
**7**	Noradechanunt et al., 2016 [26]	Australia	RCT	Older adults, n: men 3 and women 10, age: 67.6 yrs	Thai yoga, 90 min, 2/week × 12 week	30 s chair stand, 30 s arm curl, Chair sit and reach, Back scratch, 8 foot up and go, 6 min walk
**8**	Oken et al., 2006 [27]	USA	RCT	Relatively healthy adults aged 64–85, n: men 13 and women 31, age: 71.5 yrs	Ihengar yoga, 90 min, 1/week × 24 week	One leg stand, Chair sit and reach, Sit and stand, 1/4 miles walk,
**9**	Tew et al., 2018 [28]	UK	RCT	Older adults aged 60 years or older, n: women 25, age: 73.8 yrs	British Wheel yoga, 75 min, 1/week × 12 week	Standing balance, Sit to stand, 4 m walk time, Chair sit and reach, Back scratch
**10**	Tiedemann et al., 2013 [29]	Australia	RCT	Community dwelling, aged 59 years or older, n: men 5 and women 22, age: 67.7 yrs	Ihengar yoga, 60 min, 2/week × 12 week	Standing balance, sit to stand, 4 m walk time, Single leg stance eyes closed
**11**	Grossel et al., 2018 [30]	USA	RCT	Community dwelling older adults; inactive older adults, n: men 7 and women 15, age: 71.6 yrs	Ihengar yoga, 60 min, 2/week × 10 week	Chair stands, Gait speed, Balance, Grip strength
**12**	Smith et al., 2017 [31]	USA	non-RCT (pilot study)	“Community dwelling older adults, n: men 5 and women 15, age: 70.5 yrs”	Basic yoga, 60 min, 1/week × 8 week	30 s chair stand test score, FICSIT-4

Notes. RCT: randomized controlled trial; age in the table represents the mean value.

**Table 2:** Meta-analysis results—overall effect sizes and heterogeneity.

				95% CI					
Model	k	ES	SE	LL	UL	Q	df	*p*	I^2^
Fixed	57	0.455	0.029	0.399	0.511	170.339	56	0.000	67.124
Random	57	0.518	0.053	0.413	0.622				

Notes. ES: effect size; SE: standard error; 95% CI: 95% confidence interval; LL: lower limits; UL: upper limits.

**Table 3:** Sub-group analysis results—effect sizes and heterogeneity.

					95% CI					
	Items	k	ES	SE	LL	UL	Q	df	*p*	I^2^
Physical fitness	Upper limb strength	5	0.646	0.25	0.16	1.13	18.65	4	0.00	78.56
	Upper body flexibility	6	0.248	0.09	0.08	0.42	2.56	5	0.77	0.00
	Mobility	13	0.588	0.13	0.33	0.84	48.38	12	0.00	75.20
	Cardiopulmonary endurance	3	0.363	0.18	0.02	0.71	2.32	2	0.31	13.64
	Balance	12	0.639	0.15	0.35	0.93	58.48	11	0.00	81.19
	Lower limb strength	10	0.547	0.12	0.32	0.77	22.29	9	0.01	59.62
	Lower body flexibility	8	0.488	0.08	0.34	0.64	6.50	7	0.48	0.00
	Overall	57	0.460	0.04	0.38	0.54	170.34	56	0.00	67.12
Age	60s	30	0.585	0.06	0.46	0.71	66.36	29	0.00	56.30
	70s	20	0.558	0.11	0.34	0.77	79.02	19	0.00	75.95
	80s	7	0.172	0.10	−0.03	0.37	7.19	6	0.30	16.50
	Overall	57	0.487	0.05	0.39	0.58	170.34	56	0.00	67.12
Period	Under 8 weeks	22	0.543	0.07	0.40	0.69	55.98	21	0.00	62.49
	9–12 weeks	24	0.704	0.11	0.49	0.91	84.08	23	0.00	72.64
	Over 13 weeks	11	0.228	0.06	0.11	0.35	10.89	10	0.37	8.21
	Overall	57	0.415	0.04	0.33	0.50	170.34	56	0.00	67.12

Notes. ES: effect size; SE: standard error; 95% CI: 95% confidence interval; LL: lower limits; UL: upper limits.

## Data Availability

Not applicable.
